# Nasal Bridge Intramuscular Hemangioma

**DOI:** 10.1155/2015/412625

**Published:** 2015-02-02

**Authors:** Zulkifli Hamir Basah, Irfan Mohamad, Ramiza Ramza Ramli, Maha Khadum Gayadh, Samarendra Singh Mutum

**Affiliations:** ^1^Department of Otorhinolaryngology-Head & Neck Surgery, School of Medical Sciences, Universiti Sains Malaysia, Health Campus, 16150 Kota Bharu, Kelantan, Malaysia; ^2^Department of Pathology, School of Medical Sciences, Universiti Sains Malaysia, Health Campus, 16150 Kota Bharu, Kelantan, Malaysia; ^3^Taylor's University School of Medicine, 47500 Subang Jaya, Selangor, Malaysia

## Abstract

Intramuscular haemangioma (IMH) is a benign mesenchymal tumour. It appears as a deep, nontender mass within the soft tissue, particularly in the extremities. This tumour may not be obvious on clinical examination. Head and neck IMHs represent only 13.5% of the total IMHs. The most common site for a head and neck IMH is the masseter muscle, followed by trapezius, sternocleidomastoid, and very rarely temporalis muscle. We present a patient with left nasal bridge swelling which was excised and histologically confirmed as intramuscular hemangioma.

## 1. Introduction

Intramuscular haemangioma (IMH) is a rare tumour. It is benign in nature and never regresses spontaneously unlike the cutaneous hemangioma of infancy [1]. Majority of the reported cases are IMHs of the extremities in origin. In the head and neck region, it constitutes less than 20% of the total IMHs. The masseter muscle is the most frequent site in the head and neck region, accounting for 5% of all IMHs [1, 2].

## 2. Case Summary

A 22-year-old Malay man presented with a swelling over the left nasal bridge of one-year duration. The mass was slowly increasing in size but did not cause any pain. There was no history of trauma or any other nasal and eye symptoms.

**Figure 1:**
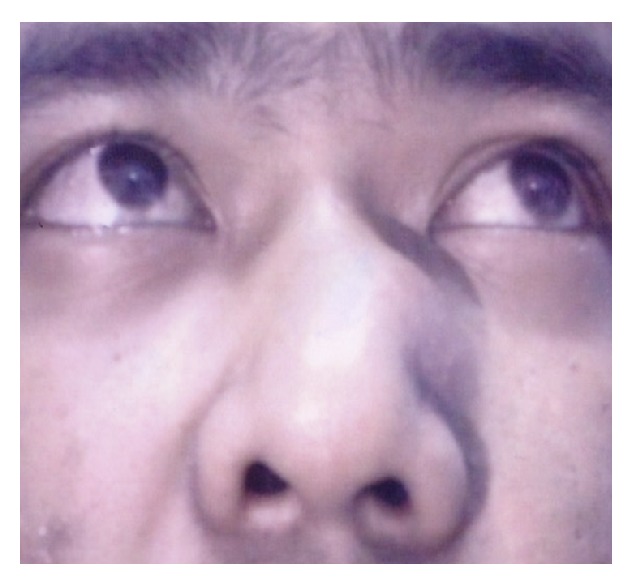
Left nasal bridge swelling.

Clinical examination revealed a swelling with the dimension of 2 × 3 cm, which was soft, nontender, and mobile in all directions (Figure 1). It was not fixed to the underlying structures or to the skin. The overlying skin and the surrounding skin were normal. There was no punctum. Nasal endoscopy revealed normal finding. Skull and nasal bone radiographs were normal. A provisional diagnosis of sebaceous cyst was made based on its clinical appearance. He underwent an excisional biopsy. Intraoperatively, there was a highly vascular mass measuring around 2 × 2 × 1 cm. There was no obvious capsule noted. The mass was excised with minimal bleeding which could be easily cauterized. The patient showed excellent recovery with minimal scar. After 6 months of follow-up, no sign of recurrence was seen.

**Figure 2:**
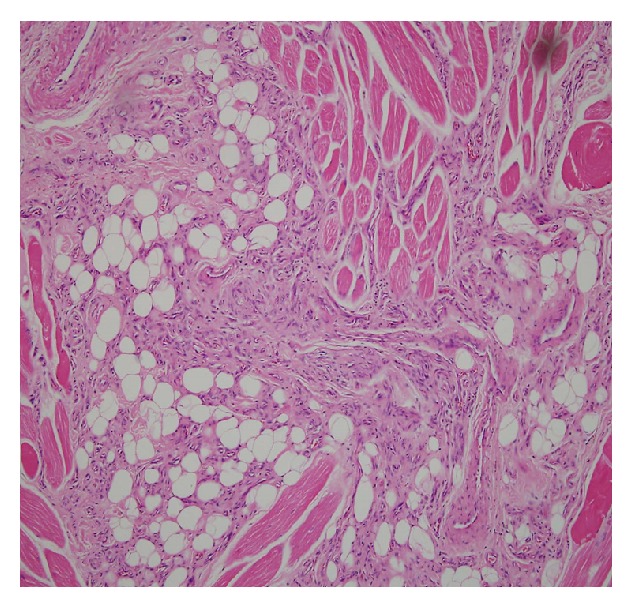
Striated muscle fibres separated by a network of predominantly thin walled capillaries with narrow lumina and scattered thick walled veins admixed with irregular lobules of adipocytes in a thin fibrocollagenous stroma are seen. Only occasional cavernous vessels are present. (H&E ×100).

The histopathological examination was reported as intramuscular hemangioma (infiltrating angiolipoma) (Figure 2). No pleomorphism or mitosis noted in the endothelial cells. Based on the findings, the diagnosis of intramuscular haemangioma of predominantly capillary types was made.

## 3. Discussion

Intramuscular hemangiomas (IMHs), which are also called infiltrating angiolipomas, are benign adipose tissue tumour that represents 5% to 17% of all lipomas in the body [3]. These tumours are hardly found in the head and neck region. Besides masseter muscle, reported head and neck IMHs sites include parotid, mandible, cheek, palate, and tongue. However, cases occurring on the external part of the nose, in particular the bridge of the nose, were rarely reported.

IMHs differ from typical lipoma and cutaneous hemangioma of infancy in that they usually arise around the time of puberty. 80% of patients will have multiple lesions and they may have a familial component [4].

Owing to its rarity in the head and neck region, the diagnosis is difficult to be obtained preoperatively. Preoperative imaging studies such as CT scan or MRI can offer a correct diagnosis and a complete resection can be performed to minimise the risk of recurrence. However, it is rarely performed in such a superficial small cystic lesion as in this indexed case. Imaging study may be indicated in the deeper seated or a bigger lesion, for example, in the IMHs of the mylohyoid or sternocleidomastoid muscle [5].

The treatment of choice for IMH, if diagnosed preoperatively, is complete wide resection of the mass including the cuff of surrounding muscle because of the infiltrative nature of the tumour [5]. Although there is a variety of treatment options, surgical resection often yields the best outcome in terms of both short term and long term results [6]. Based on histological features of the excised specimens, IMHs can be subdivided into capillary, cavernous, and mixed type according to the size of the vessels [2]. Among these subtypes, the first is more common and demonstrates the highest recurrence rate. Hence, long term follow-up is recommended.

In conclusion, IMH is a rare lesion especially in the head and neck region. Being a non encapsulated vascular neoplasm which possesses the infiltrative property. Preoperative definite diagnosis is often difficult and it may lead to inadequate resection and recurrence. A wide resection with a normal cuff of muscle should be aimed to ensure a complete cure is achieved. Long term follow-up is crucial.

## References

[1] Wolf G. T., Daniel F., Krause C. J., Kaufman R. S. (1985). Intramuscular hemangioma of the head and neck. *Laryngoscope*.

[2] Allen P. W., Enzinger F. M. (1972). Hemangioma of skeletal muscle: an analysis of 89 cases. *Cancer*.

[3] Shohet J. A., Simpson B., Coleman J. R., Geiger X. J. (1998). Angiolipoma presenting as a nasal mass. *Otolaryngology—Head and Neck Surgery*.

[4] Som P. M., Scherl M. P., Rao V. M., Biller H. F. (1986). Rare presentations of ordinary lipomas of the head and neck: a review. *The American Journal of Neuroradiology*.

[5] Lee J.-K., Lim S.-C. (2005). Intramuscular hemangiomas of the mylohyoid and sternocleidomastoid muscle. *Auris Nasus Larynx*.

[6] Cappabianca P., Cirillo S., de Divitiis E., del Basso de Caro M., Spaziante R., Zona G. (1996). Hemangioma of the temporal muscle. *Head and Neck*.

